# A New Approach for the Development of Multiple Cardiovascular Risk Factors in Two Rat Models of Hypertension

**DOI:** 10.3390/ph15070853

**Published:** 2022-07-12

**Authors:** Karyne Garcia Tafarelo Moreno, Aline Aparecida Macedo Marques, Gabriela Pereira da Silva, Bethânia Rosa Lourençone, Clara Soligo Fortini, Patrícia Regina Terço Leite, Ariany Carvalho dos Santos, Roosevelt Isaías Carvalho Souza, Leila Isabel da Siva, Arquimedes Gasparotto Junior

**Affiliations:** 1 Laboratory of Cardiovascular Pharmacology (LaFAC), Faculty of Health Sciences, Federal University of Grande Dourados, Dourados 79825-070, Brazil; karynetafarelo@gmail.com (K.G.T.M.); alinemarques_nutri@hotmail.com (A.A.M.M.); gabii.pereira6@gmail.com (G.P.d.S.); bethania.lorencone041@academico.ufgd.edu.br (B.R.L.); clarasfortini@outlook.com (C.S.F.); patricialeitesk@gmail.com (P.R.T.L.); 2 Laboratory of Histopathology, Faculty of Health Sciences, Federal University of Grande Dourados, Dourados 79825-070, Brazil; arianysantos@ufgd.edu.br (A.C.d.S.); rooseveltsouza@ufgd.edu.br (R.I.C.S.); 3 Post-Graduate Program in Biotechnology Applied to Agriculture, Paranaense University, Umuarama 87502-210, Brazil; leilaambientalmk25@hotmail.com

**Keywords:** animal models, cardiovascular disease, dyslipidemia, renal impairment

## Abstract

Cardiovascular disease (CVD) is the leading cause of death among non-communicable diseases. There is a lack of valid animal models that mimic associations among multiple cardiovascular risk factors in humans. The present study developed an animal model that uses multiple cardiovascular risk factors—namely, hypertension, hypothyroidism, and a high-fat diet (HFD). Two models of hypertension were used: renovascular hypertension (two-kidney, one clip [2K1C]) and spontaneously hypertensive rats (SHRs). The naive group was composed of normotensive rats. Twelve weeks after surgery to induce renovascular hypertension, rats in the 2K1C and SHR groups underwent thyroidectomy. The HFD was then implemented for 6 weeks. Renal function, serum redox status, biochemical CVD markers, electrocardiographic profile, blood pressure, mesenteric vascular bed reactivity, histopathology, and morphometry were investigated. Both experimental models induced dyslipidemia, renal function impairment, and hepatic steatosis, accompanied by higher levels of different inflammatory markers and serum oxidative stress. These alterations contributed to end-organ damage in all hypertensive rats. Our findings corroborate a viable alternative model that involves multiple cardiovascular risk factors and resembles conditions that are seen in humans. Both models mimicked CVD, but our data show that SHRs exhibit more significant pathophysiological changes.

## 1. Introduction

Cardiovascular disease (CVD) is the leading cause of death among non-communicable diseases globally, corresponding to an estimated 17.8 million deaths in 2017. Deaths from CVD increased by 21.1%, but death rates decreased from 259.9 to 233.1 deaths per 100,000 population in 10 years [1].

Understanding cardiovascular risk factors is fundamental for the prevention, detection, and control of CVD [2]. These risk factors include unhealthy nutrition, physical inactivity, dyslipidemia, hyperglycemia, high blood pressure, obesity, endocrine metabolic dysfunction, older age, smoking, and kidney dysfunction, with sex differences and associations with race/ethnicity [3].

Hypertension is characterized by persistently high systolic blood pressure (SBP) and diastolic blood pressure (DBP) in systemic arteries. For the outpatient diagnosis, SBP and DBP must be ≥140 and 90 mmHg, respectively [4]. Several etiologies can underlie hypertension. Most patients (90–95%) have highly heterogeneous essential hypertension with multifactorial gene–environment etiology [5]. Hypertension rarely occurs in isolation and often coexists with other cardiovascular/metabolic risk factors, such as dyslipidemia and glucose intolerance [4].

Hypertension and dyslipidemia can significantly damage the vascular endothelium, leading to impairments in vasomotor activity and creating a feedback loop that aggravates the progression of hypertension and atherosclerotic disease [6]. In addition to genetic conditions, both hypertension and dyslipidemia are possibly influenced by an unhealthy diet. Positive caloric balance and high body fat are independent risk factors for CVD. Diets with high levels of saturated fat, cholesterol, sodium, sugar, and alcohol are associated with a higher risk of CVD [3]. Nutrient metabolism and body weight can also be disturbed by changes in thyroid hormones. Thyroid hormones regulate cardiac gene expression and influence nitric oxide production and vascular tone. Thus, hypertension, dyslipidemia, and hypothyroidism constitute an important deleterious triad that impacts the cardiovascular system [7].

We identified an important gap in animal models that are useful for studying associations among multiple risk factors that are related to the genesis and exacerbation of CVD. Such models would be plausible alternatives to mimic conditions that are prevalent in human patients [8,9]. The main objective of the present study was to develop an animal model that involves multiple risk factors for CVD that are associated with hypertension, hypothyroidism, and dyslipidemia in male rats.

## 2. Results

### 2.1. Behavioral Pattern and Body Weight Gain

Behavioral patterns and body weight gain were not significantly different between the naive, two-kidney-one-clip (2K1C), and spontaneous hypertensive rat (SRH) groups (data not shown).

### 2.2. Renal Function

Urinary parameters in the different experimental groups are shown in Table 1. pH significantly decreased in all groups that were fed the high-fat diet (HFD). Urinary density significantly increased in the 2K1C group, compared with that in the naive group. Levels of urinary chloride, potassium, sodium, and creatinine significantly decreased in the 2K1C and SHR groups, compared with the levels of those parameters in the naive group.

### 2.3. Electrocardiography

Electrocardiographic parameters in the different experimental groups at the end of 6 weeks of the HFD are shown in Table 2. The SHR group exhibited significant increases in QT and QTC segments, compared with the naive group. All other electrocardiographic parameters did not change in any of the experimental groups.

### 2.4. Blood Pressure and Heart Rate

The 2K1C and SHR groups had significantly higher SBP, DBP, and mean arterial pressure (MAP) than the naive group. The 2K1C and SHR groups had significantly lower heart rates (HR) than the naive group (Table 3).

### 2.5. Mesenteric Vascular Bed Reactivity

Vascular reactivity in the mesenteric vascular bed (MVB) in the different experimental groups is shown in Table 4. All animals in the SHR group exhibited a significant reduction in the phenylephrine (Phe; 3, 10, and 30 nmol)-induced vasoconstrictor response compared with those in the naive group. All other parameters did not significantly change.

### 2.6. Blood Analyses

Alanine aminotransferase (ALT) levels significantly increased in the 2K1C group compared with its levels in the naive group (Table 5). Serum lipid levels, including triglycerides, total cholesterol, low-density lipoprotein cholesterol (LDL-C), and very-low-density lipoprotein cholesterol (VLDL-C), significantly increased in the 2K1C and SHR groups compared with the levels observed in the naive group. The SHR group had significantly higher serum total cholesterol and LDL-C than the 2K1C group (Table 5). Serum urea levels in the 2K1C and SHR groups were significantly higher than those in the naive group. The SHR group had significantly lower serum creatinine levels than the 2K1C group (Table 5).

**Figure 1:**
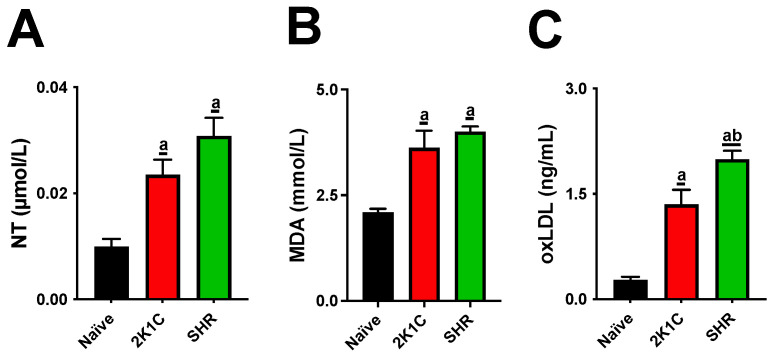
Oxidative/nitrosative stress markers in normotensive and hypertensive high-fat-fed rats. Serum nitrotyrosine (**A**), malondialdehyde (**B**), and oxidized low-density lipoprotein levels (**C**) are presented. The data are expressed as mean ± standard error of the mean. a *p* ≤ 0.05 when compared with naive; b *p* ≤ 0.05 when compared with 2K1C group. 2K1C: two-kidney-one-clip hypertension; MDA: malondialdehyde; NT: nitrotyrosine; oxLDL: oxidized low-density lipoprotein; SHR: spontaneously hypertensive rats.

**Figure 2:**
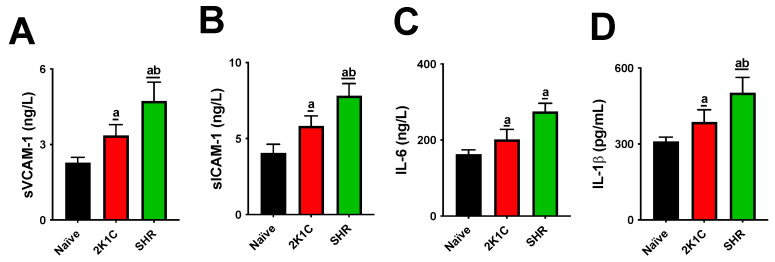
Serum interleukins and soluble adhesion molecule levels in normotensive and hypertensive high-fat-fed rats. Serum sVCAM-1 (**A**), sICAM-1 (**B**), IL-6 (**C**), and L-1β (**D**) levels are shown. The data are expressed as mean ± standard error of the mean. a *p* ≤ 0.05 when compared with naive; b *p* ≤ 0.05 when compared with 2K1C group. 2K1C: two-kidney-one-clip hypertension; IL-1β: interleukin-1β; IL-6: interleukin-6; SHR: spontaneously hypertensive rats; sICAM-1: soluble intercellular adhesion molecule-1; sVCAM-1: soluble vascular cell adhesion molecule-1.

The SHR and 2K1C groups had significantly higher nitrotyrosine (NT), malondialdehyde (MDA), and oxidized low-density lipoprotein (oxLDL) levels than the naive group. Levels of oxLDL in the SHR group were significantly higher than those in the 2K1C group (Figure 1). All rats in the SHR group had higher levels of soluble vascular cell adhesion molecule-1 (sVCAM-1), soluble intercellular adhesion molecule-1 (sICAM-1), interleukin-6 (IL-6), and IL-1β than the rats in the naive group. The SHR group also had significantly higher sVCAM-1, sICAM-1, IL-6, and IL-1β levels than the 2K1C group (Figure 2).

### 2.7. Relative Organ Weight

The SHR and 2K1C groups had significantly higher heart and liver relative weights than the naive group. The relative weight of the right kidney was not altered in any of the experimental groups (Table 6).

### 2.8. Histopathology and Morphometry

**Figure 3:**
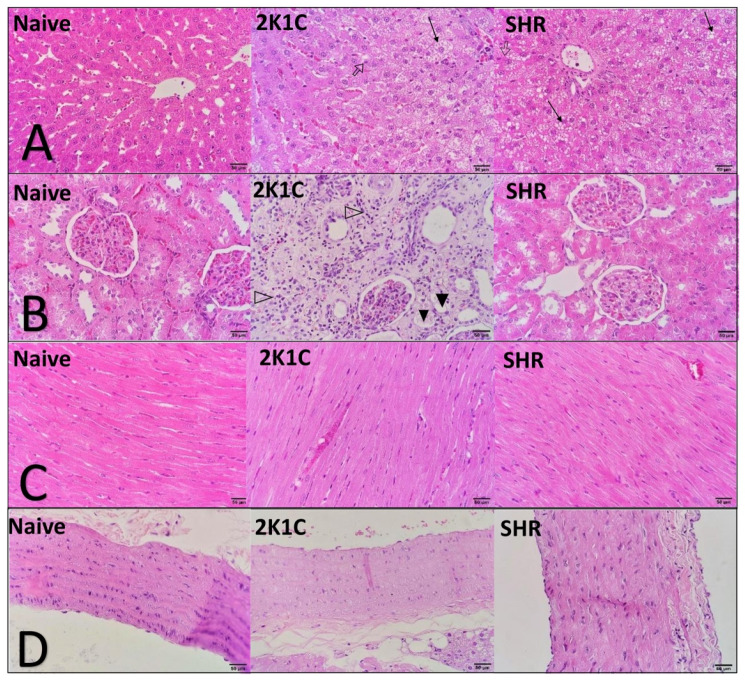
Representative histological sections of the liver (**A**), kidney (**B**), heart (**C**), and aorta artery (**D**) from normotensive and hypertensive high-fat-fed rats. The black arrows show micro and macro vacuolization and diffuse swelling of the hepatocyte’s cytoplasm. Empty-core arrows show necrosis in the liver. Empty-core arrowhead shows interstitial mononuclear inflammatory infiltrate. The black arrowhead shows thickening of the renal capsule in the remaining corpuscles and multifocal areas of calcification. H&E (40×). 2K1C: two-kidney-one-clip hypertension; SHR: spontaneously hypertensive rats.

No histopathological changes were observed in the naive group. The SHR and 2K1C groups that were fed the HFD exhibited significant liver alterations, including micro- and macrovacuolization and diffuse swelling in the cytoplasm of hepatocytes. We also observed individual cell necrosis and mononuclear inflammatory infiltrate in the liver parenchyma (Figure 3). Clipped kidneys in the 2K1C group presented a focal area with the deposition of fibrous tissue, mononuclear inflammatory infiltrate, thickening of the renal capsule, and multifocal areas of calcification. The other organs did not present significant alterations (Figure 3).

Cardiac morphometry in the different experimental groups at the end of 6 weeks of the HFD is shown in Table 6. The thickness of the interventricular septum significantly increased in the SHR and 2K1C groups, compared with that in the naive group. The 2K1C group also exhibited significant thickening of the posterior wall of the right ventricle, compared with the naive group.

## 3. Discussion

Hypertension, dyslipidemia, unhealthy nutrition, and hormonal alterations are known to strongly correlate with worse cardiovascular outcomes, either in the presence or absence of CVD [10]. Although CVD is a global health problem, animal models that combine multiple cardiovascular risk factors are scarce [11]. The present study developed an effective animal model that mimics human conditions of multiple cardiovascular risk factors. We used two classic animal models of experimental hypertension that were associated with HFD and hypothyroidism. After 6 weeks, both experimental models exhibited significant dyslipidemia, oxidative stress, and renal, vascular, and cardiac impairments that are suggestive of CVD.

The main difference between the two experimental models that were used in the present study is related to the genesis of hypertension. One model involved renovascular hypertension (2K1C), and the other model used inbred rats with spontaneous hypertension (SHR). The 2K1C model was first described by Goldblatt et al. and has become a well-established animal model of hypertension [12]. This model involves partial constriction of the renal arteries, which leads to the development of renovascular hypertension [12]. By clipping the renal artery, the renin–angiotensin system is activated, inducing the upregulation of renin release in the first weeks after surgery and leading to increases in angiotensin II and hypertension. Constriction of the renal arteries closely mimics the pathophysiology of their human analog, but renovascular hypertension represents only a small fraction of manifestations of clinical hypertension [13,14]. Spontaneous hypertensive rats are the most widely used animal model of essential hypertension. This model induces cardiac and renal impairment, without a direct relationship with chronic kidney disease [15]. The SHR strain of rats originated in Kyoto, Japan, by crossing Wistar rats that exhibited spontaneous elevations of BP. Survival of the strain was possible through inbreeding over several generations [16].

The main goal of the development of animal models is to improve approaches to prevent and treat hypertension and its complications. Hypertension is a multifactorial heterogeneous disorder, with significant variability in the pharmacokinetic/pharmacodynamic drug response. Thus, understanding this pathological variability in different animal models is fundamental for accurate and reliable preclinical trials [16]. In the present study, both experimental models exhibited dyslipidemia, renal function impairment, and hepatic steatosis, accompanied by high levels of various inflammatory markers and serum oxidative stress. Consequently, these alterations contributed to end-organ damage in all hypertensive rats.

Hypertension is associated with renal damage and progressive renal disease. High BP can disturb the autoregulation of renal capillary perfusion, thereby activating the inflammatory response [17]. Alterations in urea and creatinine levels are considered important biomarkers of renal disease progression. Urine volume and renal electrolyte excretion are also closely related to renal function [18]. In the present study, both the SHR and 2K1C groups exhibited significant decreases in urinary electrolyte excretion and increases in serum urea, compared with naive rats. Interestingly, although both experimental models significantly reduced renal creatinine clearance, only the SHR group exhibited significant elevations of serum creatinine levels. Both models of hypertension promoted renal impairment, with more pronounced changes in the SHR group.

Both experimental models significantly altered lipid metabolism, causing increases in cholesterol and triglyceride levels and LDL-C oxidation. High levels of blood lipids and oxLDL particles are thresholds for atherogenesis. IL-1β and IL-6 are cytokines that mediate the retention of oxLDL molecules in the inner layer of arteries. The expression of sVCAM-1, sICAM, and E-selectin is then stimulated, acting as adhesion molecules on the endothelial surface [19]. This process leads to the migration of monocytes to the subendothelial space and their differentiation into macrophages. Macrophages recognize oxLDL in phagocytes, the origin of foamy cells that are characteristic of atherosclerotic lesions [20]. Although our data show that both the SRH and 2K1C groups had high serum oxLDL, cytokines, and soluble adhesion molecules, these increases were higher in SHRs, suggesting a more aggressive profile for the CVD model.

Another important difference between the two models of hypertension that were used herein refers to cardiac electrical activity. QT and QTC segments were significantly higher in the SHR group than in the naive and 2K1C groups. QTC prolongation is related to a delay in ventricular repolarization and may lead to potentially life-threatening arrhythmia. QTC prolongation is often related to drug therapy, risk factors for which include old age, female sex, CVD, electrolyte abnormalities, thyroid disease, type 2 diabetes mellitus, and bradycardia [21]. In a study by Coan et al., male SHRs also exhibited higher QTC segments than normotensive control animals [22]. Similarly, all animals in the SHR group exhibited a significant reduction in the Phe-induced vasoconstrictor response compared with normotensive untreated rats. The vascular endothelium is responsible for producing several vasoactive substances, and dysfunctions in its physiology may affect important hemodynamic functions, aggravating CVD severity and evolution [23].

Histopathological and morphometric analyses showed significant changes that were induced in both experimental models of CVD. In the liver, the HFD and oxidative stress induced significant hepatic steatosis, accompanied by an increase in organ relative weight. The accumulation of dysfunctional ectopic fat in the liver is closely related to adverse cardiometabolic outcomes. The underlying mechanisms that link steatosis to CVD are complex and simultaneously involve several different pathways, including epigenetic, endothelial, inflammatory, atherogenic, metabolic, and gut microbiome factors. There is a current consensus that the presence of hepatic steatosis is an important indicator of poor prognosis for CVD [24]. In the present study, the changes in the heart were predictable in both hypertensive models, including an increase in relative weight and the thickening of cardiac chambers. These alterations worsen over the course of the disease and tend to be more severe with higher BP, thereby justifying the thickening of the right ventricle wall only in 2K1C rats (i.e., the group with the highest BP values) [10,25].

The present study had an important limitation. We were unable to identify the presence of atherosclerotic lesions in the arterial branches. Important biochemical alterations are strongly related to atherogenesis. The inability to identify atherosclerotic lesions is likely attributable to the natural resistance of rodents to short-term atherogenic factors [26]. We believe that maintaining this model for 8 or 12 weeks will lead to significant atherosclerotic lesions or contribute to other morphological changes that are characteristic of hypertensive disease.

In summary, the present study evaluated two new models of the rapid induction of heart disease in rats, which is associated with hypertension, hypothyroidism, and HFD. The present findings open new perspectives for preclinical studies of heart disease that is caused by multiple risk factors.

## 4. Materials and Methods

### 4.1. Drugs

The following drugs, salts, and solutions were used: ketamine hydrochloride (Syntec, São Paulo, SP, Brazil), xylazine hydrochloride (Syntec, São Paulo, SP, Brazil), isoflurane (BioChimico, Rio de Janeiro, RJ, Brazil), and heparin (Hipolabor, Belo Horizonte, MG, Brazil). Phenylephrine, sodium nitroprusside (SNP), acetylcholine (ACh), NaCl, KCl, CaCl_2_, MgSO_4_, NaHCO_3_, KH_2_PO_4_, dextrose, ethylenediaminetetraacetic acid, cholesterol, and colic acid were purchased from Sigma-Aldrich (St. Louis, MO, USA). All the other reagents were obtained in analytical grade.

### 4.2. Animals and Experimental Design

For this study, 12-week-old male Wistar–Kyoto rats and SHRs were housed in environmentally enriched plastic cages at 22 °C ± 2 °C, 55% ± 10% humidity, and a light/dark cycle of 12 h/12 h. These animals have ad libitum access to food and water. All experimental procedures were approved by the Institutional Ethics Committee of the Federal University of Grande Dourados (Protocol No. 10/2020) and were performed in accordance with the Brazilian Legal Framework on the Scientific Use of Animals.

**Figure 4:**
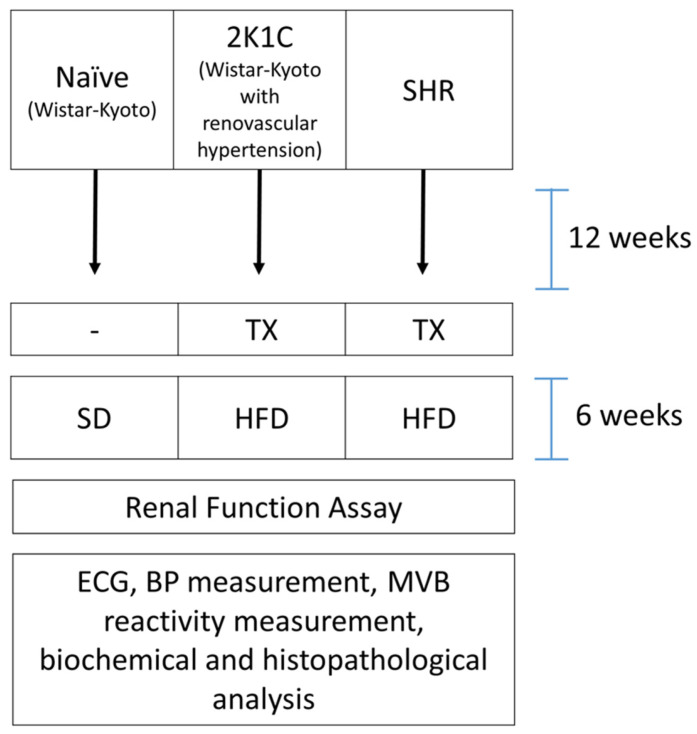
Experimental design: 2K1C: two-kidney-one-clip; HFD: high-fat diet; BP: body pressure; ECG: electrocardiography; MVB: mesenteric vascular bed; SHR: spontaneously hypertensive rats; TX: thyroidectomy.

The animals were randomized and divided into three experimental groups (*n* = 8/group): two groups of hypertensive rats (2K1C and SHR) and one normotensive group of naive rats (Figure 4).

### 4.3. Experimental Procedures

#### 4.3.1. Induction of Renovascular Hypertension

Renovascular hypertension was induced using procedures that were previously described by Ikawa et al. [27], with some modifications. Initially, the rats were anesthetized via isoflurane inhalation (1–2%) in a saturation chamber. The left renal artery was exposed by median laparotomy and dissected from the renal vein and connective tissue. A 0.25 mm lumen silver clip was attached to the left renal artery to cause partial occlusion. The muscle layer and skin were then sutured. At the end of the surgery, all animals received postoperative care for 3 days, including hydration, a sterile dressing, analgesia (0.2% meloxicam, 5 mg/kg, subcutaneously), and antibiotic therapy (10 mg/kg enrofloxacin, subcutaneously).

#### 4.3.2. Thyroidectomy

Eighty-four days after surgery to induce renovascular hypertension, the rats in the 2K1C and SHR groups underwent thyroidectomy. Total thyroidectomy was performed according to Patel et al. (2013) [28]. The rats were anesthetized by isoflurane inhalation (1–2%) and placed in the supine position. An incision (2 cm) was made in the ventral cervical midline, ending at the level of the clavicle. The underlying salivary and lymphatic glandular tissue was laterally displaced. The omohyoid muscle was dissected to visualize the trachea, larynx, and thyroid gland. The thyroid gland was gently removed, and the incision was sutured. All animals received postoperative care for 3 days, including hydration, a sterile dressing, analgesia (0.2% meloxicam, 5 mg/kg, subcutaneously), and antibiotic therapy (10 mg/kg enrofloxacin, subcutaneously).

#### 4.3.3. High-Fat Diet

The HFD was produced according to the protocol of Guarnier et al. (2019), with the following composition: 64.4% standard diet, 0.5% cholesterol, 0.1% sodium cholate, 5% sucrose, 5% lard, 5% hydrogenated fat, and 20% powdered egg [26]. Immediately after thyroidectomy, the HFD was offered to the 2K1C and SHR groups. The naive rats were fed a standard commercial diet. The diet was offered ad libitum for 42 days. 

#### 4.3.4. Renal Function Assay

On the morning of day 43, the rats were placed in metabolic cages, and urine was collected for 24 h. Urine density and pH were measured with a portable refractometer (NO107; Nova Instruments, Piracicaba, Brazil) and digital pH meter (Q400MT; Quimis Instruments, Diadema, Brazil), respectively. Urinary potassium, sodium, chloride, creatinine, and urea levels were quantified in an automated biochemical analyzer (Cobas Integra 400 plus, Roche, Basel, Switzerland).

#### 4.3.5. Electrocardiography

After urine collection, all rats were anesthetized intramuscularly with 100 mg/kg ketamine and 20 mg/kg xylazine. Four electrodes were then placed on the two forelimbs and two hindlimbs of each animal. Electrocardiography (ECG) waves were recorded for 5 min using a 12-lead ECG recorder (WinCardio, Micromed, Brasilia, Brazil) [23].

#### 4.3.6. Blood Pressure and Heart Rate Measurement

After ECG recording and while still under deep anesthesia, all animals received a subcutaneous injection of heparin (30 IU). The left carotid artery was exposed, isolated, cannulated, and connected to a pressure transducer coupled to a recording system (PowerLab) and its integration software (Chart 7.1; both from ADI Instruments, Castle Hill, Australia). Systolic blood pressure, DBP, MAP, and HR were recorded for 20 min [29].

#### 4.3.7. Mesenteric Vascular Reactivity

After direct blood pressure measurements, the MVB was isolated according to McGregor (1965) [30]. The MVB was removed, coupled to the perfusion system, and continuously perfused with physiological saline solution (119 mM NaCl, 4.7 mM KCl, 2.4 mM CaCl_2_, 1.2 mM MgSO_4_, 25.0 mM NaHCO_3_, 1.2 mM KH_2_PO_4_, 11.1 mM dextrose, and 0.03 mM ethylenediaminetetraacetic acid; aerated with 95% O_2_ and 5% CO_2_) at a constant flow rate of 4 mL/min. Changes in perfusion pressure were recorded by a pressure transducer coupled to a recording system (PowerLab) running Chart 7.1 software (both from ADI Instruments, Castle Hill, Australia). After a 30 min stabilization period, tissue integrity was assessed with a bolus injection of KCl (120 mmol). Vascular reactivity to Phe (3, 10, 30, and 100 nmol) was then evaluated. After a new 30 min stabilization period, the tissues were continuously perfused with physiological saline solution plus 3 µM Phe to induce a prolonged increase in perfusion pressure. After stabilization of the contractile process, vascular reactivity to ACh (0.3, 1, 3, and 10 nmol) and SNP (0.03, 0.1, 0.3, and 1 nmol) was evaluated.

#### 4.3.8. Blood Analyses

Immediately after MVB withdrawal, blood samples were collected from the previously cannulated left carotid artery. Serum was obtained via centrifugation at 1500× *g* for 10 min. Alanine aminotransferase, aspartate aminotransferase (AST), creatinine, urea, triglycerides, total cholesterol, and HDL-C levels were measured using a semi-automated biochemical analyzer (Aker BIO-200). LDL-C and VLDL-C were calculated according to the Friedwald formula [5]. Serum NT, oxLDL, sVCAM-1, sICAM-1, IL-1β, and IL-6 levels were measured using enzyme-linked immunosorbent assays (BD Biosciences, San Jose, CA, USA). Malondialdehyde levels were measured using an MDA assay kit (Cayman Chemical, Ann Arbor, MI, USA).

#### 4.3.9. Relative Organ Weight

At the end of the experiments, the animals were euthanized via deep anesthesia (>50–60% isoflurane) in a saturation chamber. After euthanasia, the heart, right kidney, and liver were cleaned and weighed. The relative organ weight was determined as the following:

relative weight (RW) % = absolute organ weight × 100/body weight.(1)

#### 4.3.10. Histopathology and Morphometry

Samples of the left ventricle, right kidney, liver, and subclavian and right carotid arteries were stored in 10% formalin. The material was cleaved, dehydrated in ethanol, diaphanized in xylol, and embedded in histological paraffin. The samples were sectioned (5 mm) and stained with hematoxylin–eosin and orcein (arteries) and examined under an optical microscope. The right and left ventricles and interventricular septum were measured using Motic Images Plus 2.0 Mac OS X (Kowloon Bay, Kowloon, Hong Kong).

### 4.4. Statistical Analyses

Statistical analyses were performed using one- or two-way analysis of variance (ANOVA) followed by Bonferroni’s post hoc test. The results are expressed as the mean ± standard error of the mean (SEM) of *n* = 8 animals per group. Values of *p* < 0.05 were considered statistically significant.

## 5. Conclusions

The present study identified a viable alternative to induce a combination of multiple cardiovascular risk factors in an animal model that resembles conditions that are seen in humans. Both models were able to develop significant CVD, but the SHR group exhibited more significant pathophysiological changes.

## Figures and Tables

**Table 1:** Urinary parameters of the different experimental groups at the end of 6 weeks of treatment with the high-fat diet.

Parameter	Naive	2K1C	SHR
Urinary volume (mL/100 g/24 h)	30.83 ± 6.82	23.26 ± 4.67	26.63 ± 8.00
pH	8.01 ± 0.44	7.44 ± 0.25 ^a^	7.19 ± 0.20 ^a^
Density	1015 ± 7.81	1025 ± 4.00 ^a^	1023 ± 4.42
Chloride (µmol/100 g/24 h)	1033.12 ± 121.22	657.12 ± 85.21 ^a^	700.66 ± 34.32 ^a^
Potassium (µmol/100 g/24 h)	844.21 ± 85.11	534.23 ± 56.23 ^a^	602.11 ± 45.66 ^a^
Sodium (µmol/100 g/24 h)	1311.61 ± 178.11	855.21 ± 87.34 ^a^	867.14 ± 77.66 ^a^
Urea (mg/100 g/24 h)	105.51 ± 25.22	109.12 ± 22.31	111.22 ± 30.11
Creatinine (mg/100 g/24 h)	2.66 ± 0.36	1.33 ± 0.29 ^a^	1.55 ± 0.30 ^a^

Statistical analyses were performed using one-way ANOVA followed by Bonferroni’s post hoc test. Values are expressed as standard error of the mean (mean ± SEM). *n* = 8 rats per group. ^a^ *p* ≤ 0.05 when compared with naive group. 2K1C: two-kidney-one-clip hypertension; SHR: spontaneously hypertensive rat.

**Table 2:** Electrocardiographic parameters of the different experimental groups at the end of 6 weeks of treatment with the high-fat diet.

Parameter	Naive	2K1C	SHR
P segment (ms)	31.75 ± 6.20	32.38 ± 7.05	37.33 ± 6.30
PR segment (ms)	41.00 ± 5.75	38.75 ± 10.79	44.00 ± 8.54
QRS segment (ms)	45.50 ± 4.95	54.88 ± 9.09	54.33 ± 8.68
QT segment (ms)	98.75 ± 11.56	113.50 ± 12.68	145.40 ± 16.74 ^a^
QTC segment (ms)	165.40 ± 42.19	176.40 ± 24.80	245.40 ± 36.92 ^a^
P wave (mV)	0.04 ± 0.02	0.03 ± 0.01	0.05 ± 0.01
Q wave (mV)	−0.01 ± 0.01	−0.05 ± 0.09	−0.02 ± 0.02
R wave (mV)	0.28 ± 0.05	0.25 ± 0.09	0.31 ± 0.05
S wave (mV)	−0.001 ± 0.001	−0.001 ± 0.001	−0.001 ± 0.001

Statistical analyses were performed using one-way ANOVA followed by Bonferroni’s post hoc test. Values are expressed as standard error of the mean (mean ± SEM). *n* = 8 rats per group. ^a^ *p* ≤ 0.05 when compared with naive group. 2K1C: two-kidney-one-clip hypertension; SHR: spontaneously hypertensive rat.

**Table 3:** Blood pressure and heart rates of the different experimental groups at the end of 6 weeks of treatment with the high-fat diet.

Parameter	Naive	2K1C	SHR
SBP (mm Hg)	98.60 ± 24.27	164.10 ± 24.76 ^a^	144.80 ± 10.10 ^a^
DBP (mm Hg)	63.79 ± 3.61	100.95 ± 14.50 ^a^	99.39 ± 7.86 ^a^
MAP (mm Hg)	80.22 ± 16.05	121.56 ± 12.39 ^a^	115.79 ± 8.22 ^a^
HR (bpm)	246.00 ± 11.56	178.60 ± 20.42 ^a^	153.00 ± 21.88 ^a^

Statistical analyses were performed using one-way ANOVA followed by Bonferroni’s post hoc test. Values are expressed as standard error of the mean (mean ± SEM). *n* = 8 rats per group. ^a^ *p* ≤ 0.05 when compared with naive group. 2K1C: two-kidney-one-clip hypertension; DBP: diastolic blood pressure; HR: heart rate; MAP: mean arterial pressure; SBP: systolic blood pressure; SHR: spontaneously hypertensive rat.

**Table 4:** Mesenteric vascular reactivity of the different experimental groups at the end of 6 weeks of treatment with the high-fat diet.

Parameter	Naive	2K1C	SHR
*Phe (nmol)*			
3	17.90 ± 7.92	8.96 ± 5.47	3.52 ± 1.93 ^a^
10	39.23 ± 8.07	24.90 ± 11.11	17.08 ± 7.49 ^a^
30	76.90 ± 12.13	57.38 ± 21.90	36.83 ± 9.40 ^a^
100	102.74 ± 21.98	94.66 ± 28.64	78.05 ± 31.29
*ACh (nmol)*			
0.3	−5.06 ± 1.19	−3.70 ± 1.20	−5.06 ± 1.72
1	−5.93 ± 1.90	−5.59 ± 2.05	−7.71 ± 2.98
3	−6.35 ± 2.05	−4.96 ± 2.76	−4.21 ± 1.97
10	−6.04 ± 2.66	−6.34 ± 2.18	−7.60 ± 1.54
*SNP (nmol)*			
0.03	−6.18 ± 2.52	−7.12 ± 2.18	−8.70 ± 3.56
0.1	−9.74 ± 3.39	−7.62 ± 2.50	−8.07 ± 4.33
0.3	−14.50 ± 4.82	−17.85 ± 3.13	−15.58 ± 3.81
1	−22.72 ± 8.00	−19.44 ± 6.78	−19.76 ± 6.79

Statistical analyses were performed using one-way ANOVA followed by Bonferroni’s post hoc test. Values are expressed as standard error of the mean (mean ± SEM). *n* = 8 rats per group. ^a^ *p* ≤ 0.05 when compared with naive group. 2K1C: two-kidney-one-clip hypertension; ACh: acetylcholine; Phe: phenylephrine; SHR: spontaneously hypertensive rat; SNP: sodium nitroprusside.

**Table 5:** Biochemical parameters of the different experimental groups at the end of 6 weeks of treatment with the high-fat diet.

Parameter	Naive	2K1C	SHR
AST (U/L)	193.40 ± 62.51	205.00 ± 61.02	183.40 ± 49.51
ALT (U/L)	56.00 ± 12.46	92.67 ± 10.99 ^a^	73.57 ± 11.12
Triglycerides (mg/dL)	18.75 ± 6.65	492.7 ± 143.20 ^a^	551.20 ± 160.00 ^a^
Total cholesterol (mg/dL)	89.40 ± 17.17	127.20 ± 14.96 ^a^	175.20 ± 17.03 ^ab^
HDL-C (mg/dL)	31.00 ± 10.32	28.67 ± 15.10	22.14 ± 8.97
LDL-C (mg/dL)	66.65 ± 10.64	102.33 ± 15.34 ^a^	215.40 ± 7.07 ^ab^
VLDL-C (mg/dL)	3.20 ± 1.69	98.53 ± 28.64 ^a^	123.00 ± 8.03 ^a^
Urea (mg/dL)	27.75 ± 3.59	44.33 ± 7.39 ^a^	38.60 ± 6.91 ^a^
Creatinine (mg/dL)	0.70 ± 0.21	0.90 ± 0.12	0.58 ± 0.13 ^b^

Statistical analyses were performed using one-way ANOVA followed by Bonferroni’s post hoc test. Values are expressed as standard error of the mean (mean ± SEM). *n* = 8 rats per group. ^a^ *p* ≤ 0.05 when compared with naive group. ^b^ *p* ≤ 0.05 when compared with 2K1C group. 2K1C: two-kidney-one-clip hypertension; ALT: alanine aminotransferase; AST: aspartate aminotransferase; SHR: spontaneously hypertensive rat.

**Table 6:** Relative organ weight and cardiac morphometry of the different experimental groups at the end of 6 weeks of treatment with the high-fat diet.

Parameter	Naive	2K1C	SHR
Heart (%)	0.23 ± 0.01	0.31 ± 0.02 ^a^	0.32 ± 0.02 ^a^
Right kidney (%)	0.30 ± 0.01	0.31 ± 0.06	0.32 ± 0.02
Liver (%)	2.78 ± 0.34	3.39 ± 0.16 ^a^	4.08 ± 0.30 ^a^
Right ventricle (mm)	0.78 ± 0.15	0.94 ± 0.14 ^a^	0.73 ± 0.10
Left ventricle (mm)	1.65 ± 0.20	1.76 ± 0.34	1.52 ± 0.27
IV septum (mm)	1.67 ± 0.35	2.12 ± 0.18 ^a^	2.07 ± 0.53 ^a^

Statistical analyses were performed using one-way ANOVA followed by Bonferroni’s post hoc test. Values are expressed as standard error of the mean (mean ± SEM). *n* = 8 rats per group. ^a^ *p* ≤ 0.05 when compared with naive group. 2K1C: two-kidney-one-clip hypertension; IV: interventricular septum; SHR: spontaneously hypertensive rat.

## Data Availability

The data presented in this study are available on request from the corresponding author.
